# Investigation of the relationship between atopic dermatitis of dogs and intestinal epithelial damage

**DOI:** 10.1002/vms3.1453

**Published:** 2024-04-22

**Authors:** Yusuf Emre Ekici, Mahmut Ok

**Affiliations:** ^1^ Department of Internal Medicine Faculty of Veterinary Medicine Selcuk University Konya Turkey

**Keywords:** allergens, atopic dermatitis, biomarkers, dog

## Abstract

**Background:**

A significant association between atopic dermatitis and leaky gut syndrome has been demonstrated in humans. No studies have been conducted to determine whether there is an association between atopic dermatitis and intestinal damage in dogs.

**Objectives:**

This study aimed to determine whether there is an association between canine atopic dermatitis and intestinal damage using selected intestinal‐related biomarkers.

**Methods:**

Twenty‐six dogs with atopic dermatitis and 10 healthy dogs were included. Moderate‐to‐severe pruritus, erythema, erosion and alopecia on different parts of the body were sought in dogs to suspect atopic dermatitis. The presence of atopic dermatitis was confirmed by an allergic skin test. Serum biomarkers including intestinal fatty acid binding protein (I‐FABP), intestinal alkaline phosphatase (IAP), trefoil factor‐3 (TFF‐3), immunoglobulin E (IgE), interleukin‐4 (IL‐4) and interleukin‐13 (IL‐13) concentrations were measured from venous blood samples.

**Results:**

Of the 26 dogs tested for allergens, 16 were found to be sensitive to mould mites, 10 to vernal grass, eight to house dust mites, five to wheat dust and five to grass pollen mix allergens. Significant increases in serum IAP, TFF‐3, IgE, IL‐4 and IL‐13 concentrations were determined.

**Conclusion:**

It was thought that the increase in TFF‐3 and IAP concentrations may be due to the presence of intestinal epithelial damage and the repair of this damage. In addition, the development of atopic dermatitis may be predisposed to the entry of allergens into the body through sites of intestinal damage.

## Graphical abstract

Using selected gut‐related biomarkers, this study investigated whether there is an association between canine atopic dermatitis and intestinal damage. Increased intestinal biomarkers in dogs with atopic dermatitis may indicate the presence of intestinal damage. In addition, the development of atopic dermatitis may be predisposed to the entry of allergens into the body through sites of intestinal damage.

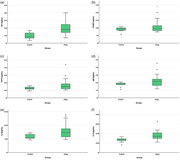

## 1 INTRODUCTION

Canine atopic dermatitis is defined as an allergic skin disease characterised by cutaneous erythema, oedema, erosions and moderate‐to‐severe pruritus. In atopic dermatitis, lesions occur on the face, concave surface of the auricle, lower abdomen, axilla, inguinal region, perineal region and distal extremities (Adam et al., 2022; Bizikova et al., 2015). Atopy is a type 1 hypersensitivity reaction of the body with immunoglobulin E (IgE) antibodies formed in the skin or in the blood circulation against environmental antigens (Maden et al., 2013; Olivry & Baumer, 2015; Pucheu‐Haston et al., 2015). Although environmental allergens such as indoor allergens, harmful dust, pollen, mould mites and grass play an important role in the aetiology of atopic dermatitis, it was reported that consumed allergenic foods also predispose to the disease (Bizikova et al., 2015; Pucheu‐Haston et al., 2015). The most common allergy is to a specific protein, but many dogs are allergic to more than one food ingredient (Shimakura & Kawano, 2021). Although contact allergens are the major contributors to the development of atopic dermatitis in dogs, food allergens are also known to predispose to the occurrence of atopic dermatitis. Foods can induce non‐immune (food intolerance) or immune (IgE‐mediated hypersensitivity) reactions (Hensel et al., 2015; Tizard, 2019).

In recent years, human medicine has shown that chronic intestinal diseases (such as irritable bowel syndrome, Crohn's disease and celiac disease) predispose to dysbiosis as a result of disruption of the intestinal microbiota or leaky gut syndrome by causing damage to the intestinal wall (Hua et al., 2016; Pascal et al., 2018; Salameh et al., 2019; Stewart et al., 2017). The entry of toxins and allergens into the organism through the tight junctions of intestinal epithelial cells that are damaged in leaky gut syndrome is thought to contribute to the development of atopic dermatitis (Kim & Kim, 2019; Tizard, 2019). Invasive histopathological methods are used to determine the location and extent of intestinal damage. Recently, intestinal damage and repair biomarkers such as I‐FABP, TFF‐3, IAP, claudin and gamma‐enteric smooth muscle actin (ACTG2) have been used as non‐invasive methods to determine intestinal epithelial damage (Durgut & Ok, 2023; Gollin et al., 1993; Ng et al., 2013; Ok et al., 2020; Shaaban et al., 2021; I. B. Yildiz & Ok, 2022; R. Yildiz et al., 2019). In the acute phase, I‐FABP, which is localised in enterocytes, enters the blood circulation as a result of cell injury/destruction (Abdel‐Haie et al., 2017) and can be used in the evaluation of intestinal injury (Gulersoy et al., 2020, 2023; Ok et al., 2020; I. B. Yıldız & Ok, 2022; R. Yıldız et al., 2018). In the chronic phase, trefoil factors (TFF‐1, 2 and 3) are peptides that play a role in the protection and repair of epithelial surfaces, including the gastrointestinal tract (Aamann et al., 2017). Also, as a result of the organism's type 1 hypersensitivity response to environmental allergens in atopic dermatitis, a significant increase in IL‐4 and IL‐13 concentrations in addition to intense IgE production has been reported (Chaudhary et al., 2019; Maden et al., 2013; Majewska et al., 2016)

In recent years, it has been established in human medicine that chronic intestinal diseases (such as irritable colon syndrome, Crohn's disease and celiac disease) predispose to dysbiosis as a result of disruption of the intestinal microbiota or leaky gut by causing damage to the intestinal wall and that this condition may form the basis of the development of atopic dermatitis. However, no studies have been conducted to determine whether there is an association between atopic dermatitis and intestinal damage in dogs. Therefore, this study has two main objectives. First, to determine if there is an association between canine atopic dermatitis and possible intestinal epithelial barrier damage (leaky gut). Second, to determine which environmental allergens commonly play a role in the aetiology of canine atopic dermatitis and, in particular, to determine whether there is an association between canine atopic dermatitis and intestinal epithelial damage using selected intestinal‐specific damage biomarkers.

## 2 MATERIAL AND METHOD

### 2.1 Animals

Twenty‐six dogs with atopic dermatitis (experimental group) and 10 healthy dogs (control group) of different breeds, aged between 1 and 14 years were included. All were admitted to the small animal clinic of the Department of Internal Medicine, Faculty of Veterinary Medicine, Selcuk University, with complaints suspecting the presence of atopic dermatitis. Favrot 2010 (Favrot et al., 2010) criteria were used to diagnose atopy, and dogs meeting five criteria are considered atopic. These criteria are: affected pinnae (but not pinnae margins), affected forelimbs, age at onset <3 years, chronic or recurrent yeast infections, predominantly indoor lifestyle, pruritus responsive to corticosteroids, unaffected dorsolumbar area, pruritus without skin lesion at onset. An allergic skin test was performed to confirm atopic dermatitis.

### 2.2 Selection of healthy dogs

The control group consisted of 10 healthy dogs that were admitted to the clinic either for vaccination or routine check‐up purposes. Animal owner's consent was obtained for each dog. Dogs whose results of routine clinical and laboratory examinations (haemogram and blood gas analysis) were within reference values and without skin lesions were considered healthy and included in the control group.

### 2.3 Selecting dogs with atopic dermatitis

The experimental group consisted of 26 dogs with atopic dermatitis. Routine clinical and dermatologic examination of dogs with skin problems and moderate‐to‐severe pruritus was performed. The dermatologic examination included an evaluation of the lesion structure of the skin. Skin scrapings were taken from the lesion sites for parasitic (scabies) and fungal (ringworm) examination. Skin scrapings were sent to the parasitology laboratory for parasitic examination. For fungal examination, the lesioned area was first examined with a Wood's lamp, and at the same time, skin scrapings were sent to the microbiology laboratory for fungal culture. To diagnose atopic dermatitis in dogs, a solution containing allergens was applied intradermally. Dogs treated with corticosteroids, cyclosporine and antihistamines were excluded from the study. The dogs with atopic dermatitis were followed and monitored throughout the treatment period.

### 2.4 Application and interpretation of allergic skin testing

To perform the allergy test, the right chest area of the dogs was carefully shaved with a clipper. No chemicals were used in the aseptic preparation of the shaved area because of the risk of an allergic reaction affecting the results. The sites to be injected with the allergen test solution were marked with a pencil at 2 cm intervals. A 0.05 mL of allergen solution was applied to the first, second, third, fourth and fifth marked areas, a negative solution was applied to the sixth marked area, and a positive control solution was applied to the seventh marked area using a sterile insulin syringe. Evaluation of allergen test results was performed 20 min after application.

Procedure for test evaluation: A ruler was used to measure the reaction diameter at the site where the Atruvetrin negative and positive control solutions were applied. The result of the negative control + the result of the positive control was divided by two (e.g., 6 mm (negative) + 12 mm (positive): 2 = 9). Allergic reactions equal to or greater than the calculated result were considered positive (Miller et al., 2012).

### 2.5 Selected allergens used to determine the aetiology of atopic dermatitis

In this study, allergens and pollen were selected because of their wide geographic variability. Selected allergen extracts are presented below.

- Grass pollen mixture allergens [1000 NU of extract of dog's tooth grass (*Cynodon dactylon*), pigweed (*Dactylis glomerata*), meadow dog tail grass (*Phleum pratense*) and velvet grass (*Holcus lanatus*) per mL)], vernal grass allergen (*Anthoxanthum odoratum*) 1000 NU extract per mL), wheat dust allergen (*Triticum aestivum*, 1000 NU extract per mL), mould mite allergen (*Tyrophagus putrescentiae*, 100 NU extract per mL), house dust mite allergen (*Dermatophagoides pteronyssinus*, 100 NU extract per mL), positive control (0.1 mg of histamine phosphate in each mL) and negative control (physiological phosphate buffer), Artuvetrin, ARTUVET Animal Health BV).

### 2.6 Blood sample collection

Blood sampling was performed via *vena saphenous* or *vena cephalica* venipuncture in all the dogs. Tubes with K_3_EDTA for haemogram and serum tubes with gel for biochemical parameter measurements were used. Haemogram measurement was performed within 15 min after sampling. Sera were stored in a deep freezer at −80°C until biomarker measurement.

### 2.7 Treatment protocol

Treatment of atopic dermatitis consisted of symptomatic treatment and diet. Prednisolone (Prednol, Mustafa Nevzat) 1 mg/kg twice daily for 10 days, cetirizine (Zyrtec, UCB Pharma) 0.5 mg/kg twice daily for 5 days, cefuroxime (Cefaks, Deva) 20 mg/kg twice daily for 10 days were given orally to dogs with atopic dermatitis. Skin lesions were washed with chlorhexidine shampoo (Diafarm chlorhexidine shampoo) twice a week for 4 weeks. Cream containing tacrolimus monohydrate (Tacrolin, Farma‐Tek) was applied to the lesion areas twice daily for 10 days. Fish oil tablets (Omepa Q10, TAB İlaç) were administered orally 1 tablet per day for 15 days. The dogs were fed with an atopic diet (Atopic Care, Advance) until the skin lesions healed. However, *Demodex* scabies was detected in three dogs with atopic dermatitis. In addition to the aforementioned treatment, the dogs received peroral ivermectin (Ivomec, Boehringer) for *Demodex* scabies at a dose of 0.1 mg/kg on Day 1, 0.2 mg/kg on Day 2, 0.3 mg/kg on Day 3, 0.4 mg/kg on Day 4, 0.5 mg/kg on Day 5, 0.6 mg/kg on Day 6 and 0.4 mg/kg on the other days for 2 months.

### 2.8 Haemogram analysis

Within the scope of haemogram analysis, white blood cells (WBC), lymphocytes (Lym), monocytes (Mon), granulocytes (Gra), eosinophils (Eos), red blood cells (RBC) and platelets (THR) were measured using MS4e device (CFE 279, Haematology Analyser Melet Schlosing Laboratories). In addition, a peripheral blood smear (Giemsa stain) was prepared for formula leukocyte count. The formula leukocyte was counted using a light microscope (x100 magnification with immersion oil).

### 2.9 Biomarkers analyses

Serum concentrations of I‐FABP, TFF‐3, IAP, IgE, IL‐4 and IL‐13 (Bioassay Technology Laboratory Co.) were measured using commercial canine‐specific Enyzme‐Linked Immunosorbent Assay (ELISA) test kits according to the manufacturer's instructions. Canine I‐FABP commercial ELISA kit (Bioassay Technology Laboratory Co., No: E0304Ca), Canine TFF‐3 commercial ELISA kit (Bioassay Technology Laboratory Co., Cat. No: E0305Ca), Canine IAP commercial ELISA kit (Bioassay Technology Laboratory Co., Cat. No: E0438Ca), Canine IgE commercial ELISA kit (Bioassay Technology Laboratory Co., Cat. No: E0208Ca), Canine IL‐4 commercial ELISA kit (Bioassay Technology Laboratory Co., Cat. No: E0003Ca), Canine IL‐13 commercial ELISA kit (Bioassay Technology Laboratory Co., Cat. No: E0351Ca) were used for ELISA analysis of biomarkers. The intra‐assay coefficient of variation (CV), inter‐assay CV and minimum detectable concentrations for the biomarkers were <8%, <12% 0.12 ng/mL for I‐FABP, <8%, <10% 0. 29 ng/mL for TFF‐3, <8%, <10% 0.26 ng/mL for IAP, <8%, <10% 0.026 µg/mL for IgE, <8%, <10% 1.02 pg/mL for IL‐4 and <8%, <10% 3.18 ng/mL for IL‐13.

### 2.10 Statistical methods

To determine the normality of the present data, the Shapiro–Wilks test was utilised. Since all variables were normally distributed, the results were presented as mean ± SEM. Independent *t* test was used to determine the difference between the groups. SPSS 22.0 for Windows package program was used for the test. Pearson's test was used to determine the correlation between variables. The significance level was accepted as *p* < 0.05.

## 3 RESULTS

### 3.1 Clinical results

In 26 dogs with atopic dermatitis, skin lesions had a distribution under the jaw, around the mouth and eyes, neck, legs, back and chest, tail, under the abdomen and at the cranial thorax. Dogs with atopic dermatitis were found to have moderate‐to‐severe pruritus, erythema, erosions and hair loss for 1 month. It was found that 20 out of 26 dogs with atopic dermatitis had diarrhoea for several (1.5–3) months. Of the 26 dogs with atopic dermatitis, 18 were fed on homemade food, seven were fed on commercial dry dog food and one was fed on chicken.

### 3.2 Allergen skin test results

**FIGURE 1:**
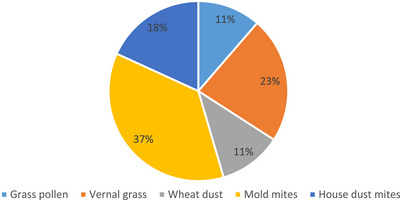
Distribution of allergen sensitisation in dogs with atopic dermatitis.

Allergen sensitivities of the dogs with atopic dermatitis are presented in Table 1 and Figure 1. Sixteen dogs were determined to be sensitive to mould mites (37%), 10 to vernal grass (23%), eight to house dust mites (18%), five to wheat dust (11%) and five to grass pollen mix (11%) allergens. It was also observed that dogs are sensitive to at least one allergen (Table 1, Figure 1).

**TABLE 1:** Sensitisation of dogs with atopic dermatitis to allergens.

Case no	Grass pollen mixture	Vernal grass	Wheat dust	Mould mite	House dust mite
1		**+**			
2				**+**	**+**
3				**+**	
4				**+**	**+**
5	**+**	**+**		**+**	
6			**+**	**+**	
7		**+**		**+**	
8				**+**	**+**
9		**+**			
10	**+**		**+**		
11			**+**		**+**
12		**+**			**+**
13				**+**	
14		**+**			**+**
15			**+**	**+**	**+**
16			**+**		
17		**+**			
18	**+**	**+**		**+**	
19	**+**				
20				**+**	
21				**+**	**+**
22				**+**	
23	**+**				
24		**+**		**+**	
25		**+**		**+**	
26				**+**	

### 3.3 Biomarker and haemogram results

**FIGURE 2:**
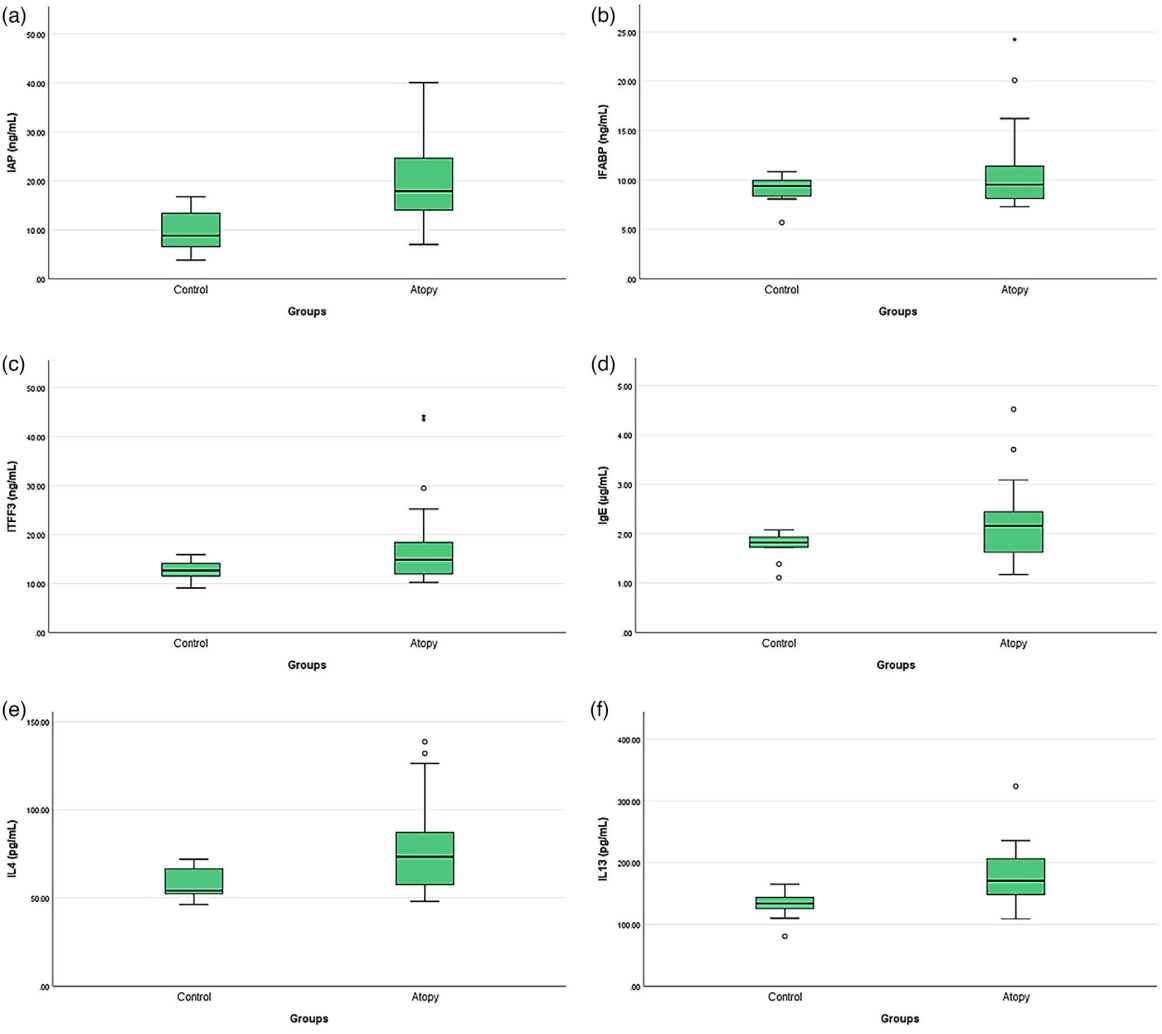
Biomarker concentrations in healthy and atopic dogs.

Biomarker concentration measurement results of the atopic and the healthy dogs are presented in Table 2. Serum concentrations of IAP (*p* < 0.01), TFF‐3 (*p* < 0.01), IgE (*p* < 0.01), IL‐4 (*p* < 0.01) and IL‐13 (*p* < 0.01) were significantly higher in dogs with atopic dermatitis, compared to the healthy ones. There was no statistically significant difference in serum I‐FABP concentrations (*p* > 0.05) between the groups (Table 2, Figure 2). While there was a statistically significant increase in Mon (*p* < 0.01) and Eos (*p* < 0.001) counts in the dogs with atopic dermatitis, compared to the healthy ones, no difference was found in WBC, Lym, Gra, RBC and THR counts (Table 2).

**TABLE 2:** Means and significance of parameters in healthy and atopic dermatitis dogs (mean ± SEM).

Parameters	Control group (*n*:10)	Experimental group (*n*:26)	*p*‐value
IAP (ng/mL)	9.56 ± 1.40	19.67 ± 1.50	0.001
I‐FABP (ng/mL)	9.08 ± 0.46	10.77 ± 0.79	0.074
TFF‐3 (ng/mL)	12.49 ± 0.67	17.50 ± 1.70	0.012
IgE (µg/mL)	1.74 ± 0.09	2.18 ± 0.14	0.015
IL‐4 (pg/mL)	58.06 ± 2.70	78.3 ± 4.80	0.001
IL‐13 (pg/mL)	131.50 ± 7.30	176.00 ± 9.10	0.001
WBC (m/mm^3^)	12.88 ± 1.27	14.73 ± 1.17	0.296
LYM (m/mm^3^)	3.63 ± 0.40	4.60 ± 0.62	0.201
MON (m/mm^3^)	0.44 ± 0.04	1.11 ± 0.22	0.007
GRA (m/mm^3^)	8.80 ± 1.16	9.01 ± 0.84	0.886
EOS (m/mm^3^)	0.02 ± 0.33	0.08 ± 0.46	0.001
RBC (m/mm^3^)	7.26 ± 0.27	7.50 ± 0.18	0.490
THR (m/mm^3^)	167.27 ± 22.75	206.16 ± 17.91	0.193

Abbreviations: EOS, eosinophils; GRAs, granulocytes; IgE, immunoglobulin E; IL‐13, interleukin‐13; IL‐4, interleukin‐4; IAP, intestinal alkaline phosphatase; I‐FABP, intestinal fatty acid binding protein; LYMs, lymphocytes; MONs, monocytes; RBC, red blood cells; THR, platelets; TFF‐3, trefoil factor 3; WBC, white blood cells.

### 3.4 Results of correlation analysis

The results of the correlation analysis between intestinal IAP, I‐FABP, TFF‐3, IgE, IL‐4, IL‐13, monocyte and eosinophil are presented in Table 3. Significant positive correlations were determined between IAP and I‐FABP, TFF‐3, IgE, IL‐4, IL‐13 (*p* < 0.01) and eosinophils (*p* < 0.05). A positive correlation was determined between I‐FABP and IAP, TFF‐3, IgE, IL‐4 and IL‐13 (*p* < 0.01). A significant positive correlation was determined between TFF‐3 and IAP, I‐FABP, IgE, IL‐4 and IL‐13 (*p* < 0.01). A significant positive correlation was determined between IgE and IL‐4 and IL‐13 (*p* < 0.01). A significant positive correlation (*p* < 0.01) was determined between IL‐4 and IL‐13 (Table 3).

**TABLE 3:** Pearson correlation analysis between serum IAP, I‐FABP, TFF‐3, IgE, IL‐4, IL‐13, monocyte and eosinophil parameters in dogs with atopic dermatitis.

Variable	IAP	I‐FABP	TFF‐3	IgE	IL‐4	IL‐13	Monocyte	Eosinophil
IAP	1	0.558^**^	0.524^**^	0.626^**^	0.458^**^	0.548^**^	0.326	0.391^*^
I‐FABP		1	0.826^**^	0.739^**^	0.727^**^	0.693^**^	−0.017	0.027
TFF‐3			1	0.761^**^	0.686^**^	0.781^**^	−0.031	0.095
IgE				1	0.571^**^	0.731^**^	0.045	0.128
IL‐4					1	0.674^**^	−0.139	0.150
IL‐13						1	0.061	0.312
Monocyte							1	0.275
Eosinophil								1

Abbreviations: I‐FABP, intestinal fatty acid binding protein; IgE, immunoglobulin E; IL‐13, interleukin‐13; IL‐4, interleukin‐4; IAP, intestinal alkaline phosphatase; TFF‐3, trefoil factor 3.

* *p* < 0.05.

** *p* < 0.01.

## 4 DISCUSSION

In this study, 16 out of 26 dogs with atopic dermatitis were determined to be sensitive to dust mites, 10 to vernal grass, eight to house dust mites, five to wheat dust, and five to grass pollen allergen. In addition, most dogs with atopic dermatitis were found to be sensitive to at least one allergen (Table 2, Figure 1). In accordance with the previous reports (Adam et al., 2022; Favrot et al., 2010; Gedon & Mueller 2015; Hensel et al., 2015), house dust mites, wheat dust, meadow and grass pollen play a role in the aetiology of the occurrence of atopic dermatitis. In the present study, it was observed that dust mites, vernal grass, grass pollen, house dust mites and wheat dust allergens play an important role in atopic dermatitis in dogs.

Atopic dermatitis in dogs is thought to be associated with exposure to house dust mite, wheat dust, meadow and grass pollen. However, allergens have been reported to cause seasonal (pollen) or non‐seasonal (mites in dust or food) atopic dermatitis (Gedon & Mueller, 2015; Hensel et al., 2015; K. Ural, 2022). In this study, mould mite allergen was detected in 16 out of 26 atopic dermatitis cases, fragrant meadow grass allergen in 10, house dust mite allergen in eight, wheat dust allergen in five, and grass pollen allergen in five. In addition, some of the atopic dogs gave reactions to one or more allergens (Table 1). In accordance with the aforementioned reports (Gedon & Mueller, 2015; Hensel et al., 2015; K. Ural, 2022), house dust mite, wheat dust, meadow and grass pollen play a role in the aetiology of atopic dermatitis. Although fragrant meadow grass, grass pollen, house dust mite and wheat dust allergens played an important role in the present study, mould mite allergens were determined to be the most prominent allergens. We believe that the probable reason why dust mite allergens caused atopic dermatitis in dogs in the study may be related to the fact that these dogs were kept in kennels and the floors of the kennels were damp. This is because wet floors are the primary environment for the reproduction of mould mites. In addition, many researchers (Gedon & Mueller, 2015; Hensel et al., 2015; K. Ural, 2022) reported that allergens can cause atopic dermatitis seasonally (pollen) or non‐seasonally (mites in dust or food). The observation of atopic dermatitis cases in April and October and the identification of pollen allergens (grass pollen mix, meadow grass and wheat dust) and mite allergens (mould and house dust mite) in the occurrence of atopic dermatitis in this study are consistent with the findings of the above‐mentioned researchers.

Although contact allergens are the major contributors to the development of atopic dermatitis in dogs, food allergens are also known to predispose to the occurrence of atopic dermatitis. Foods can induce non‐immune (food intolerance) or immune (IgE‐mediated hypersensitivity) reactions (Hensel et al., 2015; Tizard, 2019). The most common allergy is to a specific protein, but many dogs are allergic to more than one food ingredient (Shimakura & Kawano, 2021). Gastrointestinal disorder findings such as vomiting, diarrhoea, tenesmus, soft stools, flatulence and increased bowel movements have been reported in atopic dermatitis due to food allergy (Hensel et al., 2015). In this study, 18 dogs with atopic dermatitis were fed on homemade diets, one dog was fed on chicken and seven dogs were fed on commercial dry dog food. We believe that the fact that 18 dogs with atopic dermatitis were fed home‐cooked food, the fact that home‐cooked food consisted mostly of unbalanced and poor quality incompatible foods with unknown ingredients, and the reactions to these foods may have predisposed the skin to environmental allergen sensitisation. The fact that 20 of the 26 dogs with atopic dermatitis had an episode of diarrhoea several (1.5–3) months ago further strengthens our hypothesis that food reactions may contribute to the occurrence of atopic dermatitis. Additionally, this hypothesis has been supported in many studies (Hensel et al., 2015; Tizard, 2019).

In recent years, it has been established in human medicine that chronic intestinal diseases (such as irritable colon syndrome, Crohn's disease and celiac disease) predispose to dysbiosis as a result of disruption of the intestinal microbiota or leaky gut by causing damage to the intestinal wall and that this condition may form the basis of the development of atopic dermatitis (Hua et al., 2016; Pascal et al., 2018; Rostaher et al., 2022; Salameh et al., 2019; Stewart et al., 2017). As a result of the disruption of the mucosal epithelial barrier in the leaky gut, allergens and toxins are thought to pass into the organism and predispose it to atopic dermatitis (Pascal et al., 2018; Thomsen et al., 2023). Dysbiotic microbiota crossing the damaged gut barrier may play a role in the development of allergies and autoimmune and chronic diseases (Akdis, 2021). Sugita et al. (2023) reported that the gut microbiota plays a pivotal role in the pathogenesis of canine atopic dermatitis and that oral faecal microbiota transplantation may be a novel therapeutic approach to target the gut microbiota in canine atopic dermatitis. Similarly, K. Ural (2022) reported that faecal microbiota transplantation capsules were beneficial in the treatment of canine atopic dermatitis. In this study, we investigated whether there is an association between canine atopic dermatitis and intestinal epithelial barrier damage. The biomarkers I‐FABP, TFF‐3, IAP, IgE, IL‐4 and IL‐13, which indicate intestinal damage and inflammation in dogs, were evaluated. There was a statistically significant increase in serum TFF‐3 (*p* < 0.012) and IAP (*p* < 0.001) concentrations in dogs with atopic dermatitis, compared to the healthy dogs, while there was no difference in I‐FABP concentration (Table 2). Increased serum concentrations of TFF‐3 and IAP in the atopic dogs may indicate the presence of damage to the intestinal epithelial barrier. Elevated serum concentrations of IAP and TFF‐3 may indicate the possibility of the development of a leaky gut in dogs with atopic dermatitis. Because TFF‐3 and IAP biomarkers are involved in the repair and protection of the intestinal mucosal epithelial barrier, these biomarkers are intensively released from the intestine to repair the developing intestinal damage. The fact that 20 out of 26 dogs with atopic dermatitis had a history of illness with diarrhoea between 1.5 and 3 months ago may further suggest the possibility that these dogs may develop leaky gut as in humans. We suggest that toxins and allergens entering the organism through the disrupted intestinal barrier and the development of dysbiotic microbiota may have contributed to the development of atopic dermatitis in these dogs (Hua et al., 2016; Pascal et al., 2018; Rostaher et al., 2022; Stewart et al., 2017; K. Ural, 2022). The fact that D. A. Ural (2022) reported that leaky gut and intestinal permeability increased in calves with *Giardia duodenalis*‐infected diarrhoea supports our view.

It has been demonstrated that serum I‐FABP concentration significantly increases in enteritis caused by various agents in humans and animals, and this marker can be used in the diagnosis of intestinal damage (Durgut & Ok, 2023; Ng et al., 2013; Ok et al., 2020; Shaaban et al., 2021; I. B. Yildiz & Ok, 2022; R. Yildiz et al., 2019). It has been reported that I‐FABP may be an important biomarker in the early diagnosis of necrotising enterocolitis (NEC; Shaaban et al., 2021). The increase in serum I‐FABP and L‐FABP concentrations in calves with atresia coli was associated with the development of ischemic damage due to wall compression by the contents accumulated in the anterior part of the closed colon (R. Yildiz et al., 2018). On the other hand, it has been shown that there is a significant increase in serum I‐FABP concentration in people with celiac disease (Oldenburger et al., 2018). It has been reported that I‐FABP concentrations are higher in humans with inflammatory bowel disease, compared to healthy individuals (Sarikaya et al., 2015). Ok et al. (2020) showed that serum I‐FABP and L‐FABP concentrations were significantly increased in calves with diarrhoea caused by various infectious agents, and they reported that these biomarkers are important and reliable in determining intestinal epithelial damage. It was also reported that serum I‐FABP concentrations were significantly elevated in dogs with parvoviral enteritis, compared to healthy dogs, and I‐FABP is a useful marker not only in determining intestinal epithelial damage but also in predicting mortality in patients (Gulersoy et al., 2020). I. B. Yildiz and Ok (2022) found that serum I‐FABP concentration was statistically significantly higher in dogs with isosporiasis than in healthy dogs, and I‐FABP concentration decreased significantly after treatment. Durgut and Ok (2023) found a statistically significant increase in serum I‐FABP concentration in calves with coccidiosis, compared to healthy calves. In the present study, serum I‐FABP concentrations in the atopic dogs were not statistically different from the healthy dogs. However, significant positive correlations were found between I‐FABP and IAP, TFF‐3, IgE, IL‐4 and IL‐13 (*p* < 0.01; Table 3). The reason why I‐FABP concentration did not change in the present study may be related to the elevation of this biomarker in acute intestinal injury. The finding that most of the dogs with atopic dermatitis (20 cases) had enteritis 1.5 to 3 months ago can be interpreted as chronic cases. In addition, the significant (*p* < 0.01) positive correlation between I‐FABP and IAP and TFF‐3 suggests the possibility of intestinal injury.

Several researchers have reported that TFF‐3 is a peptide that plays a role in intestinal epithelial barrier repair and protection (Gulersoy et al., 2020; Ok et al., 2020; Srivastava et al., 2015; Wang et al., 2019; Yang et al., 2021; I. B. Yildiz & Ok, 2022). They demonstrated that serum TFF‐3 concentrations were higher in dogs with parvoviral enteritis, compared to healthy dogs, and its evaluation together with I‐FABP is a useful marker for predicting intestinal damage and mortality (Gulersoy et al., 2020). Ok et al. (2020) found that serum TFF‐3 concentrations were significantly increased in enteritis caused by various infectious agents in neonatal calves and TFF‐3 along with I‐FABP, L‐FABP and IAP were useful and reliable biomarkers in determining intestinal epithelial damage. In dogs with isosporiasis (I. B. Yildiz & Ok, 2022) and in calves with coccidiosis (Durgut & Ok, 2023), there was no statistical difference in serum TFF‐3 concentration, compared to healthy animals before treatment, but there was a significant increase after treatment. In the present study, serum TFF‐3 concentrations were found to be statistically significantly higher in the dogs with atopic dermatitis, compared to the healthy dogs (Table 2). Significant positive correlations were observed between TFF‐3 and IgE, IL‐4 and IL‐13 (*p* < 0.01; Table 3). The elevated TFF‐3 concentration may be related to the intensive release from goblet cells to repair the damaged epithelial barrier associated with intestinal epithelial barrier damage in dogs with atopic dermatitis. The fact that the increased serum TFF‐3 concentrations in calves with coccidiosis (Durgut & Ok, 2023) and in dogs with isosporiasis (I. B. Yildiz & Ok, 2022) were due to the intense release of TFF‐3 from goblet cells to repair the epithelial damage caused by *Eimeria* oocysts in the intestine supports the present findings. However, the significant (*p* < 0.01) positive correlations between TFF‐3 and I‐FABP and IAP suggest that these biomarkers are important diagnostic parameters in the detection of intestinal injury. The present results were consistent with the previous reports (Gulersoy et al., 2020; Ok et al., 2020; Srivastava et al., 2015; Wang et al., 2019; Yang et al., 2021; I. B. Yildiz & Ok, 2022; R. Yildiz et al., 2018).

Intestinal alkaline phosphatase is released from the apical microvilli of enterocytes into the intestinal lumen. IAP is mainly released from the duodenum and jejunum but to a much lesser extent from the ileum and colon (Malo et al., 2014). Kampanatkosol et al. (2014) reported that serum IAP levels were significantly increased in humans with NEC, and this marker can be used in the early diagnosis of NEC. An experimental study showed that IAP is an important factor in protecting against intestinal mucosal damage (Kühn et al., 2020). It was reported that IAP was significantly higher in humans with intestinal necrosis, compared to healthy individuals (McLachlan et al., 1993). It was reported that serum IAP concentrations were significantly increased in calves with atresia coli (R. Yildiz et al., 2018), calves with neonatal diarrhoea (Ok et al., 2020) and dogs with isosporiasis (I. B. Yildiz & Ok, 2022). However, Durgut and Ok (2023) reported no statistical difference in serum IAP concentration in calves with coccidiosis before and after treatment, compared to healthy calves. The lack of increase in IAP level was attributed to the damage caused by *Eimeria* oocysts in the cecum and colon where IAP release is limited. In the present study, a statistically significant increase in serum IAP (*p* < 0.001) concentration was found in the atopic dogs, compared to the healthy dogs (Table 2). In addition, significant positive correlations were determined between IAP and I‐FABP, IgE, IL‐4, IL‐13 (*p* < 0.01), and eosinophils (*p* < 0.05; Table 3). Similar to reports by many investigators in intestinal inflammations of various aetiologies (Goldberg et al., 2008; Kampanatkosol et al., 2014; Kühn et al., 2020; Molnar et al., 2012; Ok et al., 2020; I. B. Yildiz & Ok, 2022; R. Yildiz et al., 2018), our serum IAP levels were significantly increased in dogs with atopic dermatitis. The present study revealed an increase in serum IAP concentration in dogs with atopic dermatitis. The fact that the majority of dogs with atopic dermatitis (20 cases) had diarrhoea a few months ago, the intestine had not yet fully healed, and the damage to the intestinal mucosal barrier had not been repaired, may have increased the release of IAP. Since the most important role of IAP is to repair the intestinal mucosal barrier, another role is to prevent bacterial translocation in the intestine (Lalles, 2012) and to reduce inflammation in enterocolitis caused by bacterial endotoxins (Tuin et al., 2009). In the present study, it was interpreted that the increase in the concentrations of TFF‐3 (*p* < 0.012) and IAP (*p* < 0.001), which repair intestinal mucosal barrier damage in dogs with atopic dermatitis, may indicate the presence of long‐term damage in the intestine. Particularly in this study, the lack of increase in I‐FABP, a biomarker of intestinal damage in the acute phase, may confirm this. In addition, the finding of a highly significant (*p* < 0.01) positive correlation between TFF‐3 and I‐FABP with IAP may indicate that these biomarkers are important diagnostic parameters in the detection of intestinal injury.

In dogs, abundant IgE production occurs as a result of the organism's type 1 hypersensitivity response to environmental allergens such as house dust mites, mould mites, wheat dust, grass or vernal grass allergens, pollen, epidermal antigens, insect antigens and feathers, resulting in a significant increase in serum IgE levels (Fraser et al., 2003). Many researchers (Maden et al., 2013; Pucheu‐Haston et al., 2015) have reported high IgE production in house dust mite‐induced atopic dermatitis in dogs. Allergen‐specific IgE concentrations were found to be higher in environmental mite‐induced atopy (Pucheu‐Haston et al., 2015). Adam et al. (2022) and Chaudhary et al. (2019) found significant increases in serum IgE concentrations against environmental allergens in dogs with atopic dermatitis. In the present study, serum IgE concentrations of the atopic dogs were statistically increased (*p* < 0.012), compared to the healthy dogs (Table 2). However, significant positive correlations were determined between IgE and IL‐4 and IL‐13 (*p* < 0.01). The present results are in agreement with the previous reports (Adam et al., 2022; Chaudhary et al., 2019; Pucheu‐Haston et al., 2015). In the present study, elevated serum IgE concentrations in dogs with atopic dermatitis were associated with type I hypersensitivity reactions to house dust mites, mould mites, wheat dust, grass pollen and vernal grass allergens. This finding was supported by the fact that the aforementioned environmental allergens have been detected in dogs with atopic dermatitis by skin allergy testing. The significant positive correlations between IgE and IL‐4 and IL‐13 (Table 3) indicate that the immunologic response to type I hypersensitivity reaction in atopic dermatitis is caused by environmental allergens in dogs by increasing IgE, IL‐4 and IL‐13 production. In atopic dermatitis, the immune response to environmental allergens causes eosinophil degranulation, neutrophil and lymphocyte activation, and the release of some cytokines (Nemoto‐Hasebe et al., 2009). Increased concentrations of IL‐4, IL‐12, IL‐13 and IL‐18 have been found in humans with atopic dermatitis (Howell et al., 2009). Nuttall et al. (2014) reported a significant increase in IL‐4 concentrations in dogs with atopic dermatitis, while no change in IL‐6 and IL‐10. It has been reported that the specific diagnostic cytokines of atopic dermatitis are IL‐4 and IL‐13 (Chan, 2018). In dogs with atopic dermatitis, serum IL‐13 increased more than IL‐4, suggesting that IL‐13 may play an important role in the development of the atopic response, compared to IL‐4 (Majewska et al., 2016). Similarly, Majewska et al. (2016) reported that IL‐13 concentrations were significantly increased in dogs with atopic dermatitis, while IL‐4 did not change. It has been reported that IL‐17, IL‐31 and IgE concentrations are significantly increased in dogs with atopic dermatitis (Chaudhary et al., 2019). In the present study, a statistically significant increase in serum IL‐4 (*p* < 0.001) and IL‐13 (*p* < 0.001) concentrations was determined in dogs with atopic dermatitis, compared to the healthy ones (Table 2). In addition, a significant positive correlation (*p* < 0.01) was observed between IL‐4 and IL‐13 (Table 3). As previously reported (Chan, 2018; Howell et al., 2009; Nuttall et al., 2014), the increase in serum IL‐4 and IL‐13 concentrations in dogs with atopic dermatitis in this study may indicate that the cytokine response to allergens in atopic dermatitis caused by environmental allergens in dogs is mainly mediated by IL‐4 and IL‐13. Although Majewska et al. (2016) reported that IL‐13 may play an important role in the formation of atopic response, compared to IL‐4 in dogs with atopic dermatitis, in the present study, it was shown that both IL‐4 and IL‐13 cytokine responses to environmental allergens occur in dogs with atopic dermatitis. The present finding was also in agreement with Chan (2018) that the specific diagnostic cytokines of atopic dermatitis are IL‐4 and IL‐13.

In conclusion, it was demonstrated that mould mites, house dust mites, grass pollen mix, spring grass and wheat dust allergens play an important role in the aetiology of atopic dermatitis in dogs. The high concentrations of serum TFF‐3 and IAP biomarkers suggest that intestinal damage has occurred, and these markers were intensively released from the intestine to repair this damage. It was interpreted that this condition is probably related to chronic intestinal damage (leaky gut). Possibly, in the formation of atopic dermatitis, the entry of allergens into the organism from the damaged areas of the intestine may predispose the formation of atopic dermatitis.

## AUTHOR CONTRIBUTIONS

**Yusuf Emre Ekici**: Investigation; conceptualisation; validation; writing; resources; data curation; software. **Mahmut Ok**: Supervision; conceptualisation; project administration; validation; data curation; methodology; investigation; writing—original draft; writing—review and editing.

## CONFLICT OF INTEREST STATEMENT

The authors declare no conflicts of interest.

## FUNDING INFORMATION

The Selcuk University Scientific Research Projects Coordinatorship under grant number 20203011

## ETHICS STATEMENT

The authors confirm that the ethical policies of the journal, as noted on the journal's guidelines page, have been adhered to and the appropriate ethical review committee approval has been received. The study was conducted with the approval of the Selcuk University Veterinary Faculty Experimental Animal Production and Research Center Ethics Committee with decision number 2020/66.

## Data Availability

The data that support the findings of this study are openly available in [repository name e.g “figshare” at http://doi.org/[doi], reference number [reference number].
